# The exposure of Sydney (Australia) to earthquake-generated tsunamis, storms and sea level rise: a probabilistic multi-hazard approach

**DOI:** 10.1038/srep07401

**Published:** 2014-12-10

**Authors:** F. Dall'Osso, D. Dominey-Howes, C. Moore, S. Summerhayes, G. Withycombe

**Affiliations:** 1 Asia – Pacific Natural Hazards Research Group, School of Geosciences, the University of Sydney; 2 NOAA Pacific Marine Environmental Laboratory; 3 Sydney Coastal Councils Group Inc

## Abstract

Approximately 85% of Australia's population live along the coastal fringe, an area with high exposure to extreme inundations such as tsunamis. However, to date, no Probabilistic Tsunami Hazard Assessments (PTHA) that include inundation have been published for Australia. This limits the development of appropriate risk reduction measures by decision and policy makers. We describe our PTHA undertaken for the Sydney metropolitan area. Using the NOAA NCTR model MOST (*Method for Splitting Tsunamis*), we simulate 36 earthquake-generated tsunamis with annual probabilities of 1:100, 1:1,000 and 1:10,000, occurring under present and future predicted sea level conditions. For each tsunami scenario we generate a high-resolution inundation map of the maximum water level and flow velocity, and we calculate the exposure of buildings and critical infrastructure. Results indicate that exposure to earthquake-generated tsunamis is relatively low for present events, but increases significantly with higher sea level conditions. The probabilistic approach allowed us to undertake a comparison with an existing storm surge hazard assessment. Interestingly, the exposure to all the simulated tsunamis is significantly lower than that for the 1:100 storm surge scenarios, under the same initial sea level conditions. The results have significant implications for multi-risk and emergency management in Sydney.

Australia is at risk from tsunamis but due to its geographic proximity to different seismic source regions, the tsunami hazard around Australia varies significantly^1^ (Figure 1a). Since the 2004 Indian Ocean tsunami disaster great effort has been made to understand the palaeo- and historic records of tsunamis affecting Australia, estimate future tsunami probabilities and to assess risk^1^,^2^,^3^. For the New South Wales (NSW) coast, the most likely sources of earthquake-generated tsunamis are the New Hebrides, Puysegur, Kermadec, Tonga and Chile Trenches^1^.

Burbidge et al.^1^,^4^ are the only team to publish a Probabilistic Tsunami Hazard Assessment (PTHA) for Australia. Burbidge et al.^1^ estimated tsunami offshore wave amplitudes (i.e. at the -100m bathymetric contour) corresponding to various annual probabilities of occurrence (i.e. probability of a given wave amplitude to be reached or exceeded), triggered by all possible seismic sources around Australia. The methodology is based on the approach usually adopted for Probabilistic Seismic Hazard Assessments (PSAH). Burbidge et al.^1^ simulated a number of earthquakes whose probability of occurrence was known for each of the selected tsunami sources (i.e. all subduction zones surrounding Australia). Other tsunami sources such as landslides and volcanic eruptions were not considered. The vertical component of the crustal deformation of each earthquake was used as the initial condition of a finite-difference numerical model to propagate each tsunami towards the coast reaching a bathymetric contour of -100m. This produced a maximum tsunami wave amplitude (at the -100m contour) for each earthquake, associated to its probability of occurrence.

The probabilistic hazard maps of Burbidge et al.^1^ are given at an offshore bathymetric contour of -100m. Near-shore tsunami propagation and inundation modelling were not undertaken. To the best of our knowledge, no PTHAs that incorporate onshore inundation modelling have been published in Australia.

In a pilot study, Dall'Osso et al.^2^ explored the exposure and vulnerability of buildings to inundation that would be associated with a tsunami run-up of 7 m in the Sydney suburb of Manly. They showed that such a scenario would inundate 1200+ buildings. However, Dall'Osso et al.^2^ estimated the tsunami inundation using a deterministic, static “bathtub filling” method that limited the scope of their work.

Historically, tide-gauge records show that only small tsunamis have affected NSW^5^. However, according to the “Australian Megatsunami Hypothesis” (AMH) large palaeotsunamis may have occurred repeatedly throughout the Holocene although their sources remain unknown^6^. Although the AMH is controversial, the fact remains that the exposed NSW coast (Figure 1a) is home to approximately 80% of the State's population. In addition to the AMH, geological evidence of smaller tsunamis that occurred in the mid to late Holocene has been found in various estuary locations along the southeast coast of Australia^7^. Although these events would have been smaller than those proposed by the AMH, if repeated today, their consequences would be significant^8^.

In fact, Chen and McAneny^9^ estimated that in Sydney, some 20,000 properties are at risk from inundation associated with a variety of coastal processes being located <1 km from the shoreline and at no more than 3m above sea level. However, this estimate did not include tsunamis.

The deficiency of PTHAs coupled with dynamic numerical inland inundation modelling and building exposure assessments make it difficult for state and local government authorities and the emergency services to implement comprehensive multi-hazard risk reduction strategies, including to the threat posed by tsunamis. The paucity of PTHAs in Australia means that the risk from tsunamis cannot be fully quantified nor compared against natural hazards whose probabilities are known. Further, in the absence of this information, the NSW Government^10^ defines tsunami risk in broad terms as ‘the entire population living within 1 km of the shoreline, and below 10m above sea level'. This approach can under or overestimate exposure, particularly in areas featuring estuaries and ria-like coasts, such as Sydney^11^.

Our study produces a more accurate PTHA to assist and underpin coastal risk management and land-use planning strategies. In particular, this work aims to:

- Undertake a PTHA for a densely populated area in south Sydney, NSW. The study area comprises the bays of Botany and Bate, and the estuary zone of Port Hacking (Figure 1b). The PTHA includes hydrodynamic modelling of tsunami generation, transoceanic and near-shore propagation, and inland inundation. Using the model MOST (Method of Splitting Tsunamis)^12^, we simulate 36 tsunami scenarios by combining three annual probabilities of occurrence (i.e. 1/100, 1/1,000 and 1/10,000), two tsunami source locations (i.e. New Hebrides and Puysegur trenches) and six initial sea level states (accounting for tide fluctuations and sea level rise estimates). For each scenario we obtain the tsunami arrival time, the wave amplitude field, the extent of the forecast area of maximum inundation, and estimates of the maximum water level and flow velocity in each point of the study area.
- Assess the exposure of buildings and infrastructure to the selected tsunami scenarios. Exposure variations across different tsunami scenarios are then discussed and compared based on the tsunami probability, source location and initial sea level conditions;
- Demonstrate how the PTHA can be used in multi-hazard studies. This is achieved by comparing the outputs of the PTHA and the exposure assessment with a probabilistic storm surge hazard and exposure assessment undertaken by McInnes et al.^11^, which used the same initial sea level conditions.

## Results

For each tsunami scenario, MOST calculated the time series of inundation depth and flow velocity reached in each cell of the topo-bathymetric grids used as inputs. This information was used to generate the following results:

### Tsunami wave amplitude fields

**Figure 1:**
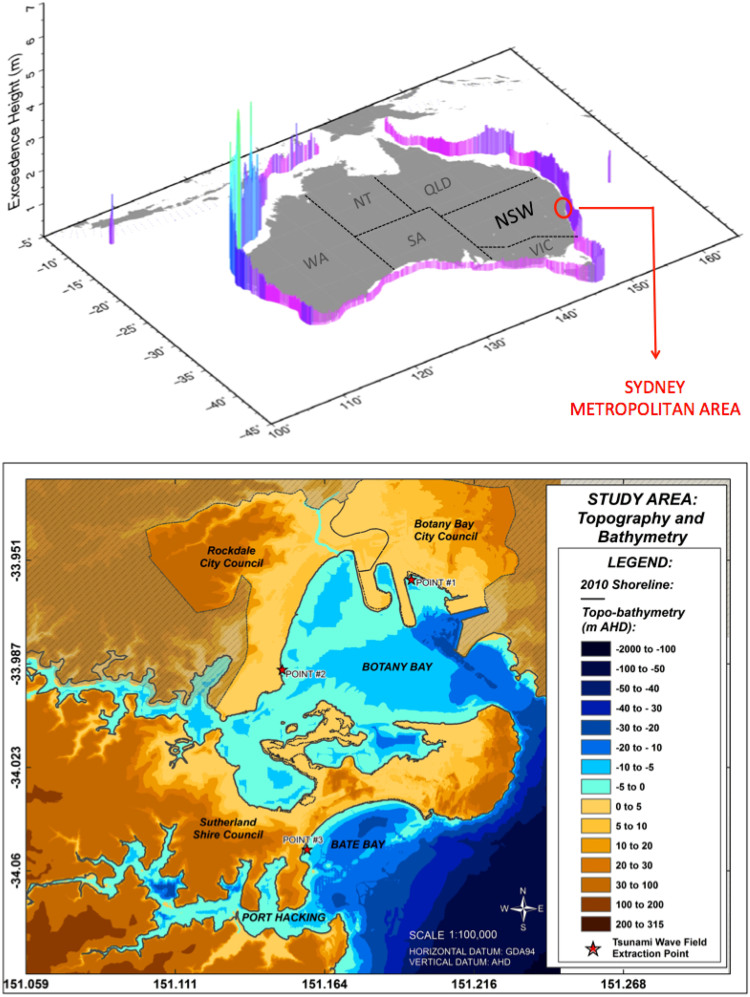
Study area and off-shore Probabilistic Tsunami Hazard Assessment for Australia. (a) the red circle indicates the study area location: this is part of the metropolitan area of Sydney, in which most of the NSW population is clustered. The colour bars surrounding Australia represent the off-shore tsunami wave amplitude having a 1 in 1,000 chance of being exceeded per year.; (b) the case study area located south of central Sydney, NSW and includes Botany Bay, Bate Bay and Port Hacking. These areas are located within the Councils of Botany Bay, Rockdale and Sutherland Shire. Points #1, #2 and #3 located by red stars were selected to extract the tsunami wave amplitude fields shown in Figures 2 and 3. The point coordinates are: point#1 (-33.9577; 151.1930), point#2 (-33.9877; 151.1490), point #3 (-34.0503; 151.1582) (Datum: CGS WGS84). Each point has a vertical elevation of -2m AHD. Figure 1(a) was modified from Burbidge *et al.*^1^, with permission from Geoscience Australia. © Commonwealth of Australia (Geoscience Australia) 2014. Figure 1(b) was created by the first author (FD) using ESRI ArcGIS 10.

**Figure 2:**
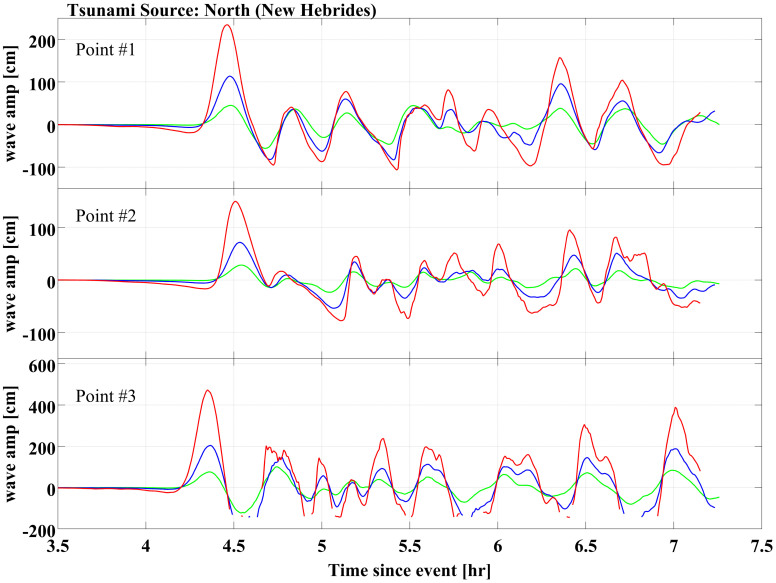
Wave amplitude fields of tsunamis triggered in New Hebrides (north of the study area), including the 1/100 (in green), the 1/1,000 (in blue) and the 1/10,000 (in red) events. The wave amplitude fields are extracted at Point #1 (Botany Bay – Sydney Airport)(Figure 2a), Point #2 (Rockdale - Ramsgate Beach) (Figure 2b) and Point #3 (Sutherland - Cronulla Beach) (Figure 2c). Figure 2 was generated by FD and CM using the software ComMIT.

**Figure 3:**
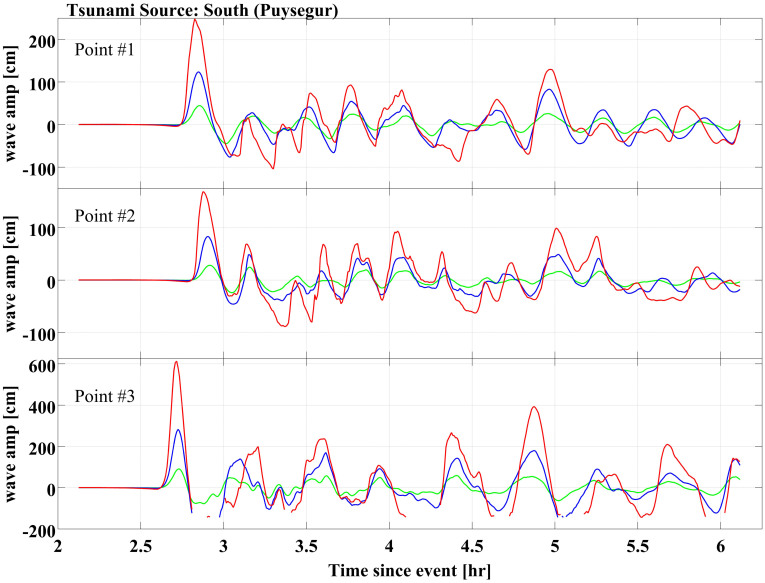
Wave amplitude fields of tsunamis triggered in Puysegur (south of the study area), including the 1/100 (in green), the 1/1,000 (in blue) and the 1/10,000 (in red) events. The wave amplitude fields are extracted at Point #1 (Botany Bay – Sydney Airport)(Figure 3a), Point #2 (Rockdale - Ramsgate Beach) (Figure 3b) and Point #3 (Sutherland - Cronulla Beach) (Figure 3c). Figure 3 was generated by FD and CM using the software ComMIT.

Examples of the tsunami wave amplitude field at three selected locations (Points #1, #2 and #3 on Figure 1b) are shown in Figure 2 and 3. As expected, the observed tsunami arrival time depends primarily on the distance between the source and the impact area (Figures 2 and 3). It is not influenced by the earthquake magnitude or by the initial sea level condition. In each scenario, multiple tsunami waves would reach the study area. In most scenarios the first wave has the largest amplitude, although there are significant exceptions. For instance, in scenario N1 (1/100 event under current sea level conditions) at Cronulla beach (Point #3, Figure 1b), the amplitudes of the second and the seventh waves would exceed the first (Figure 2c). Due to intense diffraction and reflection, locations within Botany Bay would experience more waves than ocean-facing beaches, but these waves would on average have smaller amplitudes than those impacting ocean beaches.

The first wave of each tsunami scenario originating from the Puysegur source would reach the Botany Bay entrance in about 2h30'. In this case, the first wave would have the largest amplitude, and would cause the maximum run-up. Due to the greater distance, tsunamis originating from the New Hebrides source would take about 4h20' to reach the study area. In the New Hebrides scenarios, at least seven waves would reach the study area and wave activity would last in excess of 3 hours. Significantly, at some locations, waves occurring 2 to 3 hours after the initial tsunami arrival would have amplitudes similar to the first wave.

### Inundation extent, water level and flow velocity

**Figure 4:**
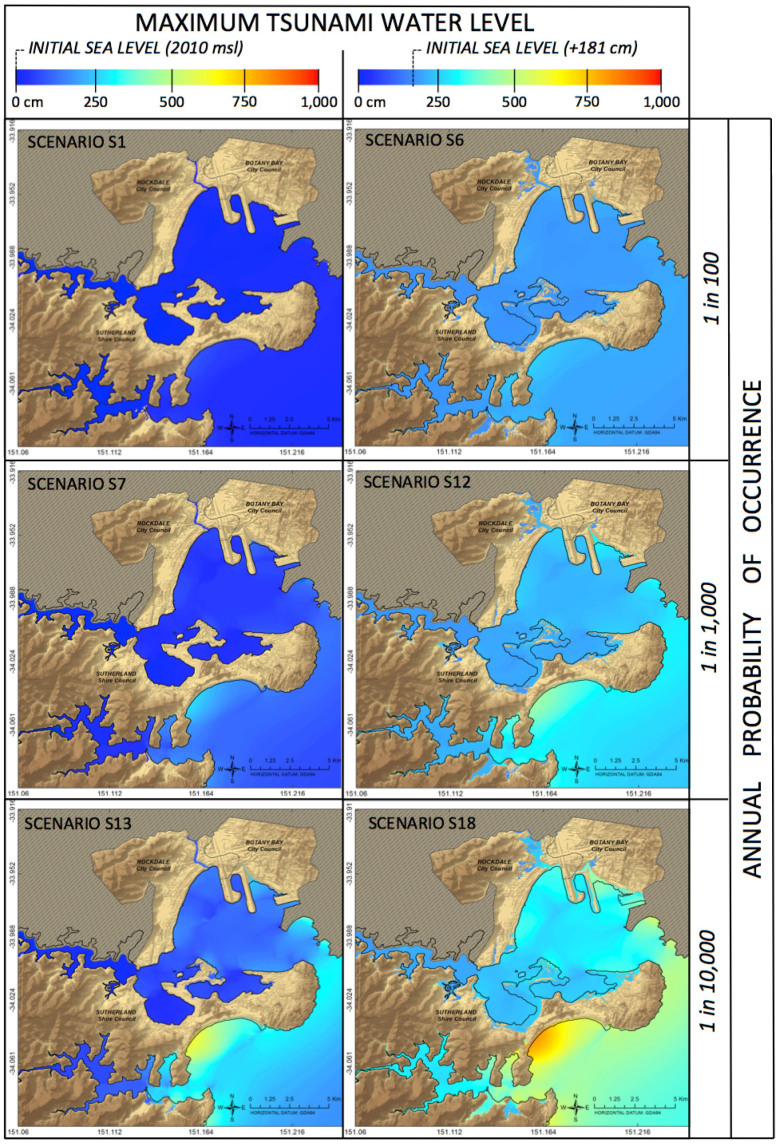
Example of a tsunami inundation maps showing the maximum water level reached during six different scenarios originating in the Puysegur Trench. Water level is measured with respect to the 2010 mean sea level. Inundation extent and water depth increase significantly with initial sea level conditions. Maps in Figure 4 were generated by FD using ESRI ArcGIS 10.

**Figure 5:**
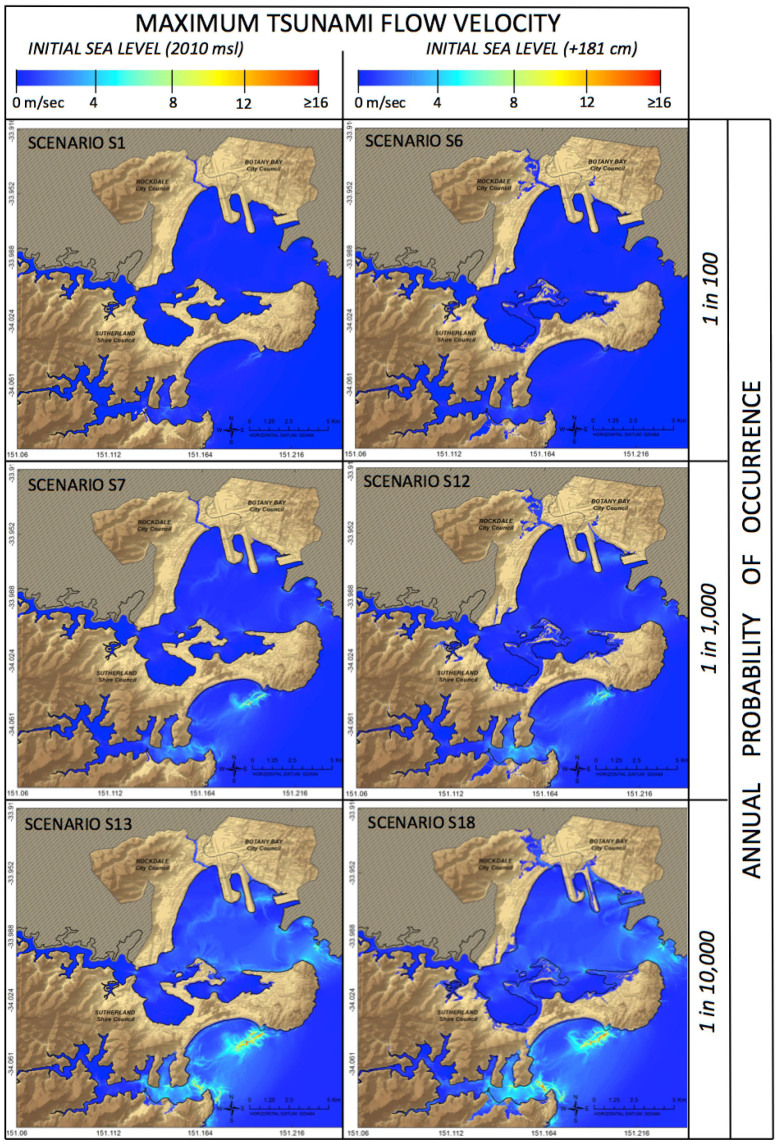
Example of a tsunami inundation map showing the maximum flow velocity (m/sec) reached during six different scenarios originating in the Puysegur Trench. Flow velocity does not depend upon the initial sea level condition but increases significantly with the tsunami intensity (i.e. inverse of the annual probability of occurrence). Maps in Figure 5 were generated by FD using ESRI ArcGIS 10.

**Figure 6:**
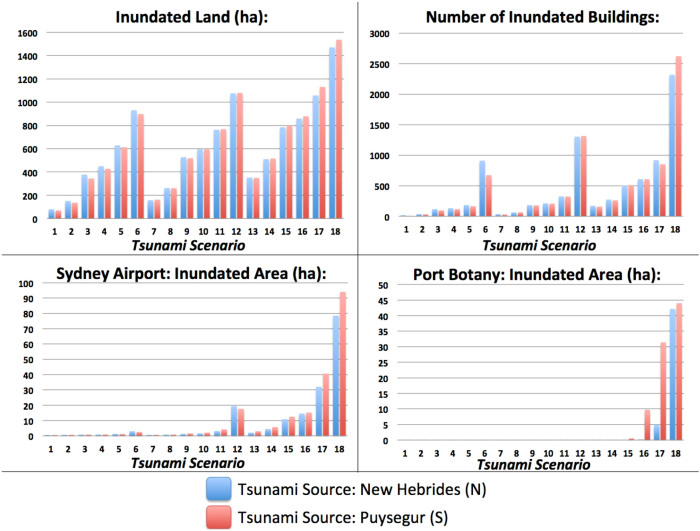
Tsunami exposure. (a) Area of land inundated by tsunami scenarios originating in the New Hebrides (scenarios N) and the Puysegur (scenarios S); (b) Number of buildings inundated in the tsunami scenarios originating in the New Hebrides and Puysegur; (c) The area of Sydney airport exposed to tsunami inundation; (d) The area of Port Botany exposed to tsunami inundation.

We generated 72 thematic Geographic Information System (GIS) maps showing the maximum inundation depth and flow velocity reached by each tsunami scenario across the study area. Example maps are shown in Figures 4 and 5. As the research generated a large number of maps it is not possible to show them all here. Interested readers may request via e-mail the entire suite of outputs including maps. These results show that for both sources events with the same annual probability cause similar inundation extents, whether triggered by the Puysegur or New Hebrides sources. The initial sea level - which increases from scenarios 1 to 6, 7 to 12 and 13 to 18 (Table 1) - has a strong influence on the extent of inundation (Figure 4 and 6) and the maximum water level.

The maximum flow velocity is mainly influenced by the tsunami probability of occurrence and not by the initial sea level condition (Figure 5). Specifically, flow velocity reaches 6.2 m/sec in the 1/100 yr. scenarios, 17.4 m/sec in the 1/1,000 yr. scenarios and over 15 m/sec in the 1/10,000 yr. scenarios (values corresponding to the 80th percentile of the flow velocity distribution, Puysegur events). These extreme values would mainly affect the entrance of Botany Bay and Port Hacking (due to a narrowing coastal morphology) and the eastern end of Bate Bay (due to an abrupt change in bathymetry) (Figure 1b). As in the case of maximum water level (Figure 4), there would be no significant difference in the flow velocity reached by events triggered by the Puysegur and New Hebrides sources.

### Exposure of buildings and infrastructure

Figure 6 shows the area of inundated land (ha) and the number of inundated buildings per scenario, including the exposure of major infrastructure such as Sydney Airport and Port Botany, the main Sydney port, both located within Botany Bay. The exposure of buildings to each tsunami scenario is relatively low under current sea level conditions, with on average 13 buildings inundated by the 1/100 yr. scenarios, 32 by the 1/1,000 yr. scenarios and 165 by the 1/10,000 yr. scenarios (Figure 6b). However, these figures increase significantly under higher sea level conditions caused by combinations of tide and/or sea level rise. In the worst-case scenarios (i.e. the 1/10,000 yr. tsunamis, with initial sea level of +181 cm above the 2010 msl, scenarios 6, 12 and 18) the average exposure peaks at 2,471 inundated buildings (Figure 6b). In fact, the exposure of buildings and infrastructure (e.g. Sydney Airport and Port Botany) shows an abrupt increase once a critical threshold of wave amplitude is attained. This happens when the initial sea level condition changes from +97-131 cm (e.g. scenarios 4 and 5) to +131-181 cm (i.e. scenarios 5 and 6) (Figure 6). This suggests that there are significant clusters of buildings located in relatively flat areas, whose elevation is only a few meters higher than the current mean sea level. Today these areas are only marginally threatened by marine inundation, but their exposure may exacerbate in the future as sea level rises.

### Comparison between tsunamis and storm surges

**Figure 7:**
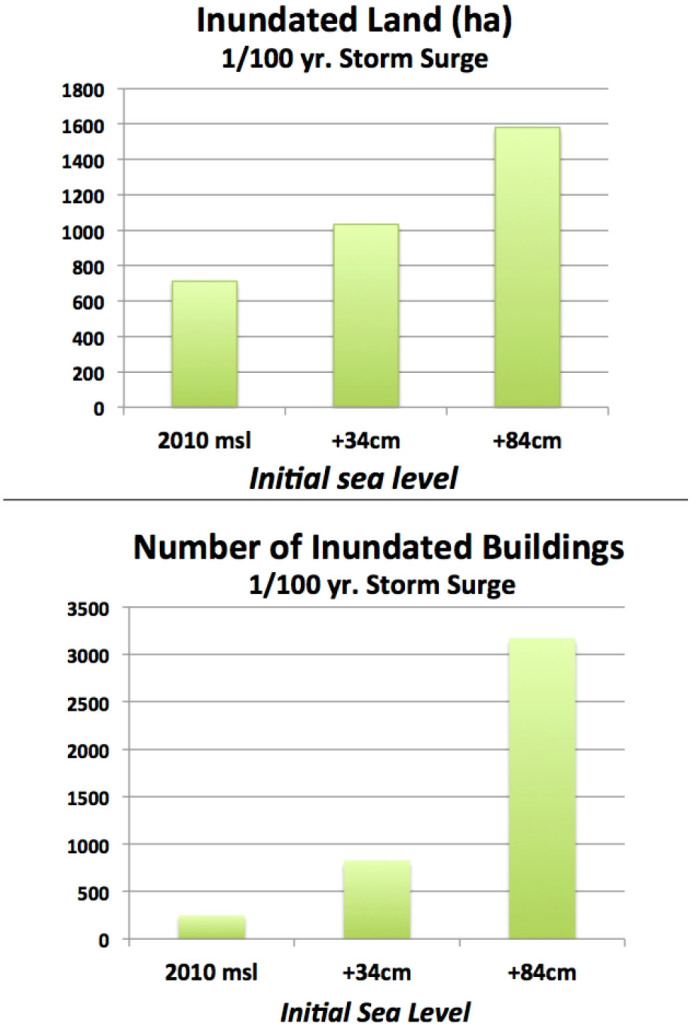
Storm surge exposure. (a) The land area inundated by the 1/100 yr. storm surge scenarios^11^ (b) Number of buildings inundated by the 1/100 yr. storm surge scenarios^11^.

An advantage of developing probabilistic tsunami hazard assessments is that they allow a comparison between different hazards with the same annual probability of occurrence. To that end, we compared the 1/100 yr. tsunami scenarios with a storm surge hazard assessment by McInnes et al.^13^. McInnes et al. simulated three storm surge events, each having an annual probability of 1/100 and occurring under three of the six initial sea level conditions used by us for the tsunami modelling (i.e. 2010 mean sea level, +34 cm and +84 cm). McInnes et al. used a combination of the wave and atmospheric conditions recorded during two past storms to generate the “design storm event” corresponding to the 1/100 yr. storm. The numerical simulation was undertaken using a combination of two different models (GCOM2D and SWAN), both widely used and validated^14^,^15^. Using this approach, McInnes et al.^11^ estimated the maximum water level along the entire Sydney metropolitan shoreline, accounting for the contributions of tide, barometrical surge and wave set-up. The inundation extent was then obtained by propagating the water level from the shoreline inland, using a static “bathtub-filling” approach. This means that all inland areas having an elevation less than the water level on the shoreline (and being hydraulically connected to it) were considered equally inundated. This process was undertaken using the same Digital Elevation Model (DEM) adopted by us. However, for the inundation part, the DEM was used at its maximum resolution of 2 m, whereas our tsunami numerical modelling used a version of the DEM resampled to a resolution of 10 m, as required by the MOST model. Our tsunami simulations considered hydraulic factors such as connectivity, storage and resistance which were not addressed by McInnes et al.^13^ to obtain the inundation extent. As a part of this study, we assessed the exposure of dry land and buildings to the three storm surge scenarios simulated by McInnes et al.^13^, within the study area (Figure 7).

Results show that:

- The total land area and the number of buildings inundated by the 1/100 storm surge event exceeds that associated with all tsunamis simulated under the same initial sea level conditions (1/100, 1/1,000 and 1/10,000 year events). This is because storm surges have a much greater frequency than tsunamis in NSW, as conducive weather conditions occur far more frequently in a 100-year time scale than tsunamis. The large difference between the inundation extent caused by storm surges and tsunamis may in part be explained by the characteristics of the storm surge numerical model, which estimates the inundated areas through a bathtub filling approach. This approach does not consider all hydraulic mechanisms controlling the inundation (such as discharge, connectivity, storage and resistance), which overestimated the inundation extent where large low-lying inland zones are connected to the sea by narrow channels or waterways. Using the modified bathtub filling approach, these areas would be completely flooded, whereas the dynamics of the process may not allow the water to physically flow through the channel until the low-lying region is filled to an equal level on the shoreline. In addition, the effect of wave run-up is not considered by McInnes et al.^13^, which may underestimate the inundation extent along ocean beaches that are directly exposed to wave action (e.g. Cronulla Beach, in Bate Bay). The tsunami numerical model (MOST) uses a hydrodynamic approach for the simulation of the whole propagation-inundation process. As a consequence, its outputs are not subject to the limitations of a bathtub-filling technique. At the same time, the spatial resolution of the tsunami inundation modelling is 10 m (whereas the storm surge inundation scenarios have a spatial resolution of 2 m). This may introduce some degree of inaccuracy at those locations where significant topographic variations occur in a relatively small area. These elements complicate a comparison with the storm surge hazard assessment by McInnes et al.^13^. As such, it is important that the differences between the two methods are borne in mind when comparing the outcomes.
- Tsunami and storm surge differ in the shape of the inundated areas. Some areas that would be flooded by 1/10,000 tsunamis would not be flooded by the storm surge scenarios. These areas are mainly located within Botany Bay and include Botany Bay Harbour, the eastern part of Sydney Airport and the urbanised area of Kurnell, at the south-eastern end of the bay; and
- There are significant differences in tsunami and storm surge inundation flow velocity. Although this parameter was not estimated by McInnes et al.^13^, previous studies show that storm surge flow velocity seldom exceeds 3–4 m/sec^16^. Significantly, the 1/100 tsunamis would generate flow velocities in excess of 6 m/sec, while 1/10,000 events would peak at over 15 m/sec. It should be noted that flow velocities higher than 11 m/sec would make safe navigation impossible^17^ and would cause significant damage to most building types, even if associated with a relatively low flow depth of 1–2 m^18^. Therefore, flow velocities up to 15 m/sec are likely to have a significant impact on the exposed coastal infrastructure and buildings.

## Discussion

This paper describes the first probabilistic tsunami hazard inland inundation assessment publicly available in Australia. The assessment includes only earthquake-generated tsunamis triggered in the Southwest Pacific (Puysegur and New Hebrides trenches). Tsunamis triggered by different mechanisms, such as localised submarine landslides or volcanic eruptions are not considered. The use of a probabilistic approach to assess tsunami hazard offers many advantages over deterministic studies. First, a probabilistic hazard assessment allows quantitative studies on risk; the probability of losing a given value (i.e. in goods and/or services) is known. This information is essential to decision makers to underpin robust cost-benefit analysis and identify cost-effective prevention and mitigation measures. In fact, low-frequency and high-consequence hazards such as tsunamis are often neglected by coastal managers because their probability of occurrence is unknown. In NSW, for example, there is no express legislative obligation for Local Government to consider tsunamis in coastal risk management, whereas other hazards such as storms and floods must be considered. In addition, probabilistic hazard assessments allow comparative multi-risk analysis (e.g. tsunamis vs. storm surges) and simplify discussions on resources allocated to coastal risk reduction strategies. This is demonstrated in this paper by comparing the exposure of buildings to tsunami and storm surge scenarios having the same annual probability of occurrence and simulated under the same sea level conditions.

Results showed that existing exposure to earthquake-generated tsunamis is relatively low, but would increase significantly under higher sea level conditions caused by combinations of tide and/or sea level rise. This reinforces the need for long-term risk reduction strategies based on the best available estimates of sea level rise. Interestingly, exposure to the 1/100 storm surges would be significantly higher than that for each tsunami scenario (1/100, 1/1,000 and 1/10,000) occurring under the same sea level conditions. Although this can be partly explained by the different numerical modelling techniques adopted in the two studies, these results reflect the fact that storm surges in NSW have a higher frequency than tsunamis, at least in the 100-year time horizon. However, although storm surges may lead to larger inundation extents, tsunamis would possess greater flow velocities, which would result in more damage to exposed buildings and infrastructure.

In addition to assessing the exposure of coastal assets, our work has important implications for emergency management, particularly evacuation considerations. The risk posed by each tsunami scenario to beachside and low-lying coastal populations would be very high. The simulations of tsunamis triggered in Puysegur showed that the first large wave would reach the study area only 2h30' after the earthquake, leaving a relatively short evacuation times.

Finally, it is important to stress that our results are subject to the following assumptions and limitations: (a) the digital elevation model (DEM) used for the tsunami numerical simulations has a horizontal resolution of 10 m and represents a “bare-earth” model, without buildings or high-rise infrastructure; (b) a single friction coefficient, derived from Manning's formula for open channel flow, is used for all dry land; (c) only earthquake-generated tsunamis were considered. Different types of tsunamis, such as those triggered by volcanic activity or by submarine landslides along the NSW continental slope, could reach the study area in a much shorter time and create different - and potentially catastrophic - inundation profiles^8^,^19^; (d) although extensively validated both with gauge and inundation data^20^,^21^, MOST is a 2D model and as such it may carry some limitations in simulating tsunami generation (via earthquakes) and inundation^22^. Future work should consider the use of 3D numerical models as these are fully validated and are adopted as international tsunami modelling standard; (e) this study incorporates the best available estimates of seismic tsunamigenic sources in the south Pacific region, currently used by the Joint Australia Tsunami Warning Centre. Future research should repeat the numerical simulation as improved plate boundary dynamics data for the Australia-Pacific region become available.

## Methods

### The study area

Our South Sydney case study area includes Botany Bay, Bate Bay and Port Hacking estuary (Figure 1b), falling within the Local Government Areas (LGAs) of Botany Bay, Rockdale and part of the Sutherland Shire. We selected this location for its high socio-economic significance as it includes the Sydney Airport and a major commercial harbour, Port Botany. Further, the Australia Department of Climate Change national risk assessment describes the area as being highly vulnerable to sea level rise and marine hazards^23^.

Botany Bay is a semi-enclosed oceanic embayment that opens to the Tasman Sea through a 1.1km-wide, southeast-facing entrance bound by bedrock cliffs. Elsewhere the bay is fringed by low-lying sandy environments, and has undergone extensive anthropogenic modifications over the last 150 years. Most of the shoreline within the bay is highly urbanised and protected by beach nourishment, seawalls and groins. The northern part of the bay has seen the development Sydney Airport and Port Botany. Botany Bay is also the location of several chemical and mining industry facilities, densely populated residential and commercial areas, well-frequented beaches and several marinas. In the southeast of the bay, a 1.1 km long pier housing a high-pressure oil pipeline extending from the Kurnell Refinery towards the centre of the bay, where a tanker loading station is located.

Bate Bay is a wide coastal embayment facing southeast. The eastern side of the bay is relatively undeveloped whereas the western end comprises the dense residential and commercial centre of Cronulla. Port Hacking (Figure 1b) is a tide-dominated ria-like estuary area of the Hacking River^24^ and is entirely located within the LGA of Sutherland Shire. While the north shore is densely populated with residential units, the south shore is mostly undeveloped and includes natural areas comprising parts of the Royal National Park^25^.

### Selecting the tsunami scenarios

We selected tsunami scenarios with annual probabilities of 1/100, 1/1,000 and 1/10,000 because they include the return time usually adopted in land use planning in Australia (1/100)^26^. Further, the 1/10,000 event underpins the draft tsunami evacuation plans by the NSW State Emergency Service (SES) (NSW SES, personal communication).

We simulated two events per annual probability, originating from (a) New Hebrides Trench (east of New Caledonia, northeast of the study area); and (b) Puysegur Trench (south of New Zealand, southeast of the study area). These were selected because of their geographic position (southeast and. northeast of our study area) and because they have the highest probabilities of triggering tsunamis with the selected annual probabilities for NSW^1^.

The tsunami scenarios were simulated under different sea level conditions to account for current and future sea level rise. We adopted the former NSW sea level rise benchmarks for AD2050 and AD2100^27^, that were based on the IPCC scenario A1FI^28^, corrected with estimates of local sea level variation for NSW^29^. These are an increase of +34 cm for AD2050 and +84 cm for AD2100, referenced to the 2010 mean sea level (msl). In this paper, we referenced the predicted sea level rise estimates to the 2010 msl^13^. We also considered the following sea level states caused by tide and surge:

- The mean sea level (no short-term sea level fluctuations)
- An increase of +97 cm above the mean sea level, corresponding to a tide and surge peak having an ARI (Average Recurrence Interval) of 0.02 yr. (i.e. about once per week)^30^.

The combination of these four factors (annual probability, source location, sea level rise, tide and surge) generates 36 different tsunami events (Table 1).

### Numerical simulation of tsunami generation, propagation and inundation using ComMIT and MOST

The numerical simulation of the tsunami scenarios was undertaken using the MOST (Method for Splitting Tsunamis) model^12^, accessed through the ComMIT (Community Model Interface for Tsunamis) platform^31^,^32^,^33^,^34^,^35^. MOST simulates earthquake-generated tsunamis via integrating the nonlinear shallow-water equations in a three-step process that includes tsunami generation, transoceanic propagation, and inland inundation. Input data include: (a) the amount and distribution of the sea-floor dislocation induced by a seismic event; (b) off-shore low-resolution bathymetry for the propagation phase; (c) a set of three high-resolution nested Digital Elevation Models (DEM) containing bathymetry and topography for use during the inundation phase (i.e. grid A, B and C). These have spatial resolutions increasing from the outer “Grid A” (32.4 arc-second), to the middle “Grid B” (3.6 arc-second), to the inner “Grid C” (0.4 arc-second), enabling the model to fully resolve the incoming wave at the nest-grid boundaries. The eastern boundary of Grid A extends off the shelf to 5,149 m depth to ensure accurate boundary conditions are transferred from the propagation phase.

While the open ocean bathymetric data required for the propagation are available through the ComMIT platform, the nested DEMs for the inundation must be manually entered. In this study, Grid A was obtained from the 9 arc-second (~260 m) national bathymetric grid created by Geoscience Australia. Grids B and C were obtained from a topo-bathymetric “bare-earth” DEM of the study area, developed by McInnes et al.^13^ by merging LiDAR data, multi-beam and echo sounding surveys. The extent of the innermost grid is shown in Figure 1b, and has a spatial resolution of 10 m and a vertical accuracy ranging between 0.15 and 0.25 m.

The ComMIT initial conditions include the location and the number of the source fault planes triggering the tsunami, the magnitude of the earthquake and a set of model parameters (e.g. time step, friction coefficient). In order to identify the initial conditions capable of triggering tsunamis with the selected annual probabilities (i.e. 1/100, 1/1,000 and 1/10,000), we ran tests adjusting the earthquake magnitude and the number of fault planes to meet the offshore wave amplitude condition estimated by Burbidge et al.^1^ for the NSW coast (Table 2). The initial conditions we used are shown in Table 3. In regards to friction during the inundation phase, overland flow velocities are limited by a Manning coefficient of 0.03, based on surface roughness typical of the "bare-earth" DEM used in this study^36^.

**Figure 8:**
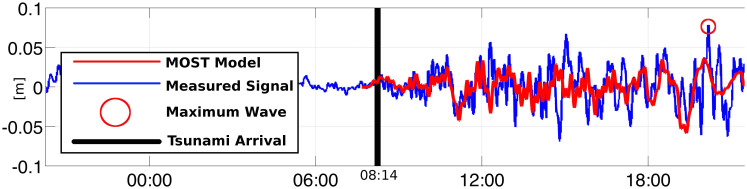
Validation of the model settings used in the study against the wave amplitude field measured by the Port Botany tide gauge during the 2010 Chile tsunami. Figure 8 was modified from Garber et al., 2011^39^, with permission from S. Garber.

Notwithstanding the limitations associated with 2D tsunami numerical models^22^,

MOST and ComMIT have been extensively benchmarked^37^, and, as MOST is in use operationally at NOAA, are validated both with tide gauge data^33^,^38^ and field-survey inundation data^7^,^8^. We validated our DEM and model setting by simulating the 2010 Chile tsunami and comparing the outcomes with the actual Botany Bay tide-gauge records of the event^39^ (Figure 8). Even if the wave signal for this event is very small (maximum peak recorded is 7 cm, about 2 hours after the beginning of the signal), Figure 8 shows that our simulation resolved the measured wave amplitude field reasonably well. The maximum wave amplitude is underestimated by about 3 cm, but this is fully consistent with the maximum vertical accuracy of the DEM (i.e. 15 cm).

### Exposure assessment

The exposure of dry land to inundation was assessed by importing the outputs of the tsunami numerical model into a GIS and intersecting the inundation layers with a DEM representing the topography of the study area. Individual buildings were manually digitized in the GIS using recent high-resolution aerial imagery provided by relevant Councils, and ground-truthed during field surveys. The number of buildings inundated by each scenario were obtained through spatial intersections with the GIS layers representing inundation extent.

## Author Contributions

F.D. undertook the research, wrote the main manuscript text and prepared all figures and tables. D.D.H. supervised the research work and the writing of the manuscript. C.M. provided significant support with the tsunami numerical simulations. S.S. managed the research project. G.W. provided guidance and access to data. All authors reviewed the manuscript.

## Figures and Tables

**Table 1:** The tsunami scenarios adopted in this study. To facilitate interpretation, a code is assigned to each scenario. The initial letter specifies the location of the tsunami source with respect to the study area (N = north of the study area, S = south of the study area). Numbers identify initial sea level conditions and the tsunami annual probability

TSUNAMI EVENT		INITIAL SEA LEVEL CONDITION			
Tsunami Source Location	Annual Probability for NSW	*Sea Level Rise (cm with respect to the 2010 sea level)*	*Tide & Surge (cm)*	Total Sea Level (cm with respect to the 2010 sea level)	Scenario Code
**New Hebrides**	**1/100**	*0*	*0*	**0**	**N1**
		*34*		**34**	**N2**
		*84*		**84**	**N3**
		*0*	*97*	**97**	**N4**
		*34*		**131**	**N5**
		*84*		**181**	**N6**
	**1/1,000**	*0*	*0*	**0**	**N7**
		*34*		**34**	**N8**
		*84*		**84**	**N9**
		*0*	*97*	**97**	**N10**
		*34*		**131**	**N11**
		*84*		**181**	**N12**
	**1/10,000**	*0*	*0*	**0**	**N13**
		*34*		**34**	**N14**
		*84*		**84**	**N15**
		*0*	*97*	**97**	**N16**
		*34*		**131**	**N17**
		*84*		**181**	**N18**
**Puysegur**	**1/100**	*0*	*0*	**0**	**S1**
		*34*		**34**	**S2**
		*84*		**84**	**S3**
		*0*	*97*	**97**	**S4**
		*34*		**131**	**S5**
		*84*		**181**	**S6**
	**1/1,000**	*0*	*0*	**0**	**S7**
		*34*		**34**	**S8**
		*84*		**84**	**S9**
		*0*	*97*	**97**	**S10**
		*34*		**131**	**S11**
		*84*		**181**	**S12**
	**1/10,000**	*0*	*0*	**0**	**S13**
		*34*		**34**	**S14**
		*84*		**84**	**S15**
		*0*	*97*	**97**	**S16**
		*34*		**131**	**S17**
		*84*		**181**	**S18**

**Table 2:** Offshore wave amplitude conditions corresponding to the tsunami annual probability of occurrence in NSW^1^

Tsunami Annual Probability of Occurrence	Offshore Wave Amplitude (depth of -100 m) in NSW	Most Likely Source Locations
1/100	23 ± 5 cm	New Hebrides, Tonga, Puysegur
1/1,000	81 ± 5 cm	New Hebrides, Puysegur
1/10,000	193 ± 5 cm	Puysegur, New Hebrides

**Table 3:** Initial conditions used in the model MOST for the simulations of the tsunami scenarios

Source Location	Annual Probability of Occurrence	Earthquake Magnitude	Slip (m)	Rupture Length (km)	Scenario Codes
New Hebrides	1/100	8.6	7.43	300	N1 to N6
	1/1,000	9.0	17.74	500	N7 to N12
	1/10,000	9.31	43.13	600	N13 to N18
Puysegur	1/100	8.3	2.64	300	S1 to S6
	1/1,000	8.65	6.62	400	S7 to S12
	1/10,000	9.05	15.6	700	S13 to S18
